# A call for collaboration on respectful, person-centered health care in family planning and maternal health

**DOI:** 10.1186/s12978-017-0280-y

**Published:** 2017-02-02

**Authors:** Kelsey Holt, Jacquelyn M. Caglia, Emily Peca, James M. Sherry, Ana Langer

**Affiliations:** 1 Women and Health Initiative, Department of Global Health and Population, Harvard T.H. Chan School of Public Health, 651 Huntington Avenue, FXB Building 7th Floor, Boston, 02115 MA USA; 2 USAID|Translating Research into Action (TRAction) Project, University Research Co., LLC, 7200 Wisconsin Avenue, Suite 600, Bethesda, 20814 MD USA; 3 USAID|Translating Research into Action (TRAction) Project, Professor of Health Policy and Management at the Graduate School for Public Health and Health Policy City University of New York, 55 W 125th St, New York, NY 10027 USA

**Keywords:** Family planning, Maternal health, Interpersonal relations, Patient centered care, Collaboration, Quality of health care

## Abstract

**Background:**

Striking tales of people judged, disrespected, or abused in reproductive, maternal, newborn, child, and adolescent health (RMNCAH) services are commonly exchanged among friends and families throughout the world while remaining sorely under-addressed in global health. Disrespect and abuse of individuals and providers in health services across the RMNCAH continuum must be stopped through collaborative, multi-tiered efforts.

**Call for collaboration:**

A new focus on health care quality in the Sustainable Development Goals offers an opportunity to seriously reexamine user experiences and their impact on health care utilization. The new framework provides an opening to redress the insidious problem of negative interactions with care across the RMNCAH services continuum and redraft the blueprint for service delivery and performance measurement, placing individuals and their needs at the center. Both the maternal health and family planning fields are at a turning point in their histories of defining and addressing individuals’ experiences of care. In this commentary, we review these histories and the current state-of-the-art in both fields. Though the approaches and language in each sub-field vary, person-centered care principles related to the essential role of individuals’ preferences, needs and values, and the importance of informed decision-making, respect, privacy and confidentiality, and non-discrimination, are integral to all. Promoting respectful, person-centered care also requires recognizing the factors that lead to poor treatment of clients, including gender norms and unsupportive working conditions for providers. Lessons can be learned from innovative efforts across the continuum to support health care providers to provide respectful, person-centered care.

**Conclusion:**

Efforts in the maternal health and family planning fields to define respectful, person-centered care provide a useful foundation from which to connect across the continuum of RMNCAH services. Now is the time to creatively work together to develop new approaches for promoting respectful treatment of individuals in all RMNCAH services.

## Background

Striking tales of people judged, disrespected, or abused in reproductive, maternal, newborn, child, and adolescent health (RMNCAH) services are commonly exchanged among friends and families throughout the world while remaining sorely under-addressed in global health. Aadhya, a married woman with three children, is scolded by her doctor for not consenting to be sterilized following her latest delivery and then pressured into using an intrauterine device (IUD). The doctor says, “Shame on you for not knowing when it’s time to stop bringing children into this world.” Patience, a teenager seeking care after a self-induced abortion, is not offered pain medication during post-abortion treatment; her provider says, “This is your punishment for having sex.” Maria, a woman of a very low socio-economic status, is slapped during labor and told to “Stop screaming like a baby.” These health care providers themselves have often been failed by systems whose infrastructure did not provide them with decent working conditions and compensation or training to enhance their communications skills. While Aadhya’s, Patience’s, and Maria’s clinical experiences differ in terms of context and content, their interactions with the health system are influenced by societal norms that devalue women’s and other disadvantaged groups’ roles in society and diminish their autonomy and humanity. Disrespect and abuse of individuals and providers in health services across the RMNCAH continuum must be stopped through multi-tiered efforts.

### A call for collaboration in family planning and maternal health care

A new focus on health care quality in the Sustainable Development Goals (SDGs) offers an opportunity to seriously reexamine user experiences and their impact on health care utilization. The new framework provides an opening to redress the insidious problem of negative interactions with care across the RMNCAH services continuum and redraft the blueprint for service delivery and performance measurement, placing individuals and their needs at the center. Negative patient experiences at any point in the service continuum can lead to delays and avoidance of care in the future, which can increase the risk of death and disability for individuals and their families. While health programs can often become siloed, it is important to remember that people and their RMNCAH needs are inextricably linked.

Both the maternal health and family planning fields are at a turning point in their histories of defining and addressing individuals’ experiences of care. This also presents an opportunity for coordinated efforts to improve care along the RMNCAH continuum. The seminal quality of care in family planning framework put forth by Judith Bruce in 1990 included a strong focus on communication and interpersonal relationships between providers and women [1]. This framework continues to underpin much of today’s work to measure and promote quality in contraception and other reproductive health services. Despite a long history of focus on quality in family planning services, the field has seen an acceleration of efforts to refine and operationalize key concepts related to experiences with care in the last few years. Following the watershed 2012 London Summit on Family Planning, the World Health Organization (WHO) and others published several new frameworks and guidance documents that detail the necessary elements of rights-based family planning and include a focus on individuals’ experiences [2, 3]. A focus on experience with contraception care is critical given the persistence of numeric targets for *uptake* of methods rather than availability of and access to high quality services, despite a push to move away from numeric targets since the 1994 International Conference on Population and Development.

In maternal health, the predominant focus has been on getting more women to give birth in health facilities. Yet facilitating more institutional births has not always translated into expected reductions in maternal mortality (especially among certain populations). Many have argued that the prevalence of poor conditions and low-skilled or over-taxed providers at many facilities are contributing factors to this shortcoming [4]. Unlike in the family planning field, a call for consistent definition and measurement of women’s experiences began fairly recently in the maternal newborn health community. The USAID|TRAction Project commissioned a landscape analysis which proposed seven categories of disrespect and abuse in facility-based childbirth in 2010 [5]. Informed by these newly proposed categories, the White Ribbon Alliance for Safe Motherhood launched the respectful maternity care charter outlining the rights of childbearing women.^1^ This was followed by a WHO statement on the prevention and elimination of disrespect and abuse during childbirth,^2^ a new model of manifestations of disrespectful and abusive treatment of women in childbirth at various levels [6], a revised typology of mistreatment of women during childbirth [7] and WHO standards for improving maternal newborn health care quality [8]. Though the approaches and language in each sub-field of RMNCAH vary, person-centered care principles related to the essential role of individuals’ preferences, needs, and values, underpin all of these frameworks. The importance of informed decision-making, respect, privacy, confidentiality, and non-discrimination are integral to all of RMNCAH.

Promoting respectful, person-centered care requires recognizing the factors that lead to poor treatment of clients, including gender norms and unsupportive working conditions for providers. *The Lancet* Commission on Women and Health recently highlighted the need to address these root causes of poor quality of care [9]. For example, approaches to guide health care providers through reflection on and articulation of their values related to care for pregnant women have long been used to increase access to safe abortion care. More recently, two approaches that support maternal health providers demonstrated potential to reduce disrespect and abuse during facility-based childbirth. The Population Council’s Heshima Project in Kenya implemented ‘Values Clarification and Attitudes Transformation’ exercises designed to help providers understand the drivers of their behavior.^3^ ‘Caring for the Carers’ debriefing sessions addressed providers’ psychosocial needs induced by work-related stress. A similar effort was undertaken in Tanzania in which the Health Workers for Change curriculum was used to promote respectful care in an urban hospital in Dar es Salaam.^4^

## Conclusion

With increasing momentum for universal health coverage (UHC) in the SDGs, a concern for promoting respectful, person-centered services, and comprehensive quality of care must come to the forefront of initiatives to improve health across individuals’ reproductive lives. UHC will only be attained if enough attention is paid to experiences of care. This includes addressing what the WHO in its statement on disrespect and abuse during childbirth deems the “violation of trust” that occurs on a daily basis when individuals are treated poorly in RMNCAH services. Efforts in the maternal health and family planning fields to define respectful, person-centered care provide a useful foundation from which to connect across the continuum of RMNCAH services. A recent review of family planning quality measurement using an emerging framework for disrespect and abuse in maternal health services provides a useful example of such collaboration [10].

Aadhya, Patience, and Maria are counting on continued collaboration across RMNCAH siloes to define and operationalize person-centered care concepts, ensure consistent measurement, and creatively work together to develop new approaches for promoting respectful treatment of individuals in all RMNCAH services.

## Abbreviations

IUD: Intrauterine device
RMNCAH: Reproductive, Maternal, Newborn, Child, and Adolescent Health
SDG: Sustainable Development Goal
UHC: Universal Health Coverage
WHO: World Health Organization

## Footnotes

1 For more information on this campaign, see here: http://whiteribbonalliance.org/campaigns2/respectful-maternity-care/

2 To view the statement, view here: http://www.who.int/reproductivehealth/topics/maternal_perinatal/statement-childbirth/en/

3 For more information on this project, see here: http://www.popcouncil.org/research/heshima-promoting-dignified-and-respectful-care-during-childbirth

4 For more information on this project, see here: https://www.mhtf.org/2016/07/25/%EF%BB%BF%EF%BB%BF-promoting-respectful-maternity-care-in-tanzania/
